# Diagnostic value of alternative techniques to gadolinium-based contrast agents in MR neuroimaging—a comprehensive overview

**DOI:** 10.1186/s13244-019-0771-1

**Published:** 2019-08-23

**Authors:** Anna Falk Delgado, Danielle Van Westen, Markus Nilsson, Linda Knutsson, Pia C. Sundgren, Elna-Marie Larsson, Alberto Falk Delgado

**Affiliations:** 1 Clinical neurosciences, Karolinska Institutet, Stockholm, Sweden; 2 Department of Neuroradiology, Karolinska University Hospital, Eugeniavägen 3, Solna, Stockholm, Sweden; 3 Department of Clinical Sciences/Radiology, Faculty of Medicine, Lund University, Lund, Sweden; 4 Department of Medical Radiation Physics, Lund University, Lund, Sweden; 5 Russell H. Morgan Department of Radiology and Radiological Science, School of Medicine, Johns Hopkins University, Baltimore, MD USA; 6 Department of Radiology, University of Michigan, Ann Arbor, MI USA; 7 Department of Surgical Sciences, Radiology, Uppsala University, Uppsala, Sweden

**Keywords:** Non-contrast-enhanced, Gadolinium, Area under curve, Diagnostic performance, Brain

## Abstract

Gadolinium-based contrast agents (GBCAs) increase lesion detection and improve disease characterization for many cerebral pathologies investigated with MRI. These agents, introduced in the late 1980s, are in wide use today. However, some non-ionic linear GBCAs have been associated with the development of nephrogenic systemic fibrosis in patients with kidney failure. Gadolinium deposition has also been found in deep brain structures, although it is of unclear clinical relevance. Hence, new guidelines from the International Society for Magnetic Resonance in Medicine advocate cautious use of GBCA in clinical and research practice. Some linear GBCAs were restricted from use by the European Medicines Agency (EMA) in 2017.

This review focuses on non-contrast-enhanced MRI techniques that can serve as alternatives for the use of GBCAs. Clinical studies on the diagnostic performance of non-contrast-enhanced as well as contrast-enhanced MRI methods, both well established and newly proposed, were included. Advantages and disadvantages together with the diagnostic performance of each method are detailed. Non-contrast-enhanced MRIs discussed in this review are arterial spin labeling (ASL), time of flight (TOF), phase contrast (PC), diffusion-weighted imaging (DWI), magnetic resonance spectroscopy (MRS), susceptibility weighted imaging (SWI), and amide proton transfer (APT) imaging.

Ten common diseases were identified for which studies reported comparisons of non-contrast-enhanced and contrast-enhanced MRI. These specific diseases include primary brain tumors, metastases, abscess, multiple sclerosis, and vascular conditions such as aneurysm, arteriovenous malformation, arteriovenous fistula, intracranial carotid artery occlusive disease, hemorrhagic, and ischemic stroke.

In general, non-contrast-enhanced techniques showed comparable diagnostic performance to contrast-enhanced MRI for specific diagnostic questions. However, some diagnoses still require contrast-enhanced imaging for a complete examination.

## Key points

- Gadolinium-based contrast agent injection should be used cautiously in neuroimaging
- Non-contrast-enhanced MRI techniques can depict specific relevant physiological processes
- MRI diffusion, arterial spin labeling, spectroscopy, and amide proton transfer imaging are non-contrast-enhanced MRI techniques that can be used to answer specific clinical questions in neuroimaging

## Background

Gadolinium-based contrast agents (GBCAs) in MRI were introduced in clinical practice in the 1980s to increase lesion detection and improve the disease characterization for many cerebral and vascular pathologies investigated with MRI [1]. The effect of GBCAs in MRI is rendering a higher signal on T1-weighted images due to shortening of T1 relaxation time. In addition, GBCA was occasionally used for patients unsuitable for iodine-based contrast agent in CT examinations.

The use of GBCAs was initially considered safe without reported acute adverse events or long-term risks. However, after several reports on the association between nephrogenic systemic fibrosis and GBCA exposure in patients with renal impairment, some linear GBCAs were withdrawn from the market or their indication was restricted [2]. More restrictive use of GBCAs in patients with renal impairment and the introduction of more stable non-linear GBCA chelates have lowered the incidence of nephrogenic systemic fibrosis [3, 4].

Recently, gadolinium deposition was been reported in the deep gray matter of the brain [5, 6] and in the bone [7]. Neither of these reports demonstrated any association with clinical symptoms. Current recommendations of the use of GBCA in clinical practice and research now emphasize that GBCAs per standard practice should only be used when absolutely necessary and that GBCA can be used in research settings with appropriate guidance of protocols and ethical review board approval with informed patient consent [5]. Furthermore, contrast enhancement adds to the cost of the MRI examination due to the cost of the additional scan time and of the contrast agent itself.

In the light of technological advances, additional costs, and possible long-term risks of GBCAs, the current role of GBCAs in neuroimaging can be discussed and re-evaluated. Only a limited number of studies have been designed to compare the diagnostic efficacy for non-contrast-enhanced MRI compared to contrast-enhanced techniques [8–10]. The aim of this paper is to review the potential use of non-contrast-enhanced MRI instead of contrast-enhanced MRI for specific diagnostic questions in neuroradiology. We describe the mechanism and limitations of GBCA contrast enhancement as well as the techniques that may serve as alternatives to GBCA contrast enhancement. Briefly, the topic of dynamic contrast-enhanced MRI is discussed. Then, we compare the diagnostic value and clinical applicability of non-contrast-enhanced with contrast-enhanced sequences in ten common diseases affecting the brain and report relevant statistical measures regarding diagnostic performance. Disease categories were ordered by the level of evidence for the ability of non-contrast-enhanced MRI to replace contrast-enhanced MRI in a clinical setting. The discussion gives a critical appraisal of the main findings.

## Search strategy

Eligible articles were searched for in PubMed until September 2018. Search strings included a combination of the following search terms: MRI, MR, AUC (area under curve), sensitivity, specificity, diagnostic accuracy, performance, gadolinium, contrast, enhanced, and GBCA. Searches were conducted by two of the authors (AnFD, AlFD).

## GBCAs and contrast enhancement

It is unclear how GBCA is taken up and is eliminated from the brain [11]. Tight junctions in the vessel endothelium and other specific mechanisms exclusive for the blood-brain barrier are supposed to prevent large molecules such as those in GBCAs to enter the brain [12]. However, disease processes may break down the integrity of the blood-brain barrier, and these processes are predominantly inflammatory, infectious, or malignant [13]. Hence, in such lesions, GBCAs will leak through the blood vessels and result in increased signal on T1-weighted images compared to normal unaffected brain. However, since several different disease processes disrupt the integrity of the tight junctions and lead to contrast enhancement, finding contrast enhancement on brain MRI is unspecific and can sometimes be misleading in clinical decision-making.

## Limitations of static contrast enhancement in neuroimaging

While contrast enhancement in neuroimaging is used clinically to increase sensitivity to detect an abnormality, the specificity is often lower. For example, static contrast enhancement alone cannot discriminate between which are low-grade and high-grade tumors in adults and children [14, 15], pseudoprogression and true intracranial tumor progression after radiation therapy [16], and pseudoresponse and true response after anti-angiogenic or immunotherapy [17]. Correct definition of the treatment response is important in order to select the optimal treatment for the patient and avoid premature cessation of an effective treatment [17]. Further examples are discrimination between a ring-enhancing metastasis and glioblastoma [18], or abscess, or indication of low tumor grade in vascularized oligodendroglial tumors [19]. Another example is that contrast enhancement fails to detect tumor cells infiltrating beyond the contrast-enhancing lesion and into the surrounding white matter, like in high-grade gliomas [20]. Further, the diffusion time of GBCA is not often considered in clinical practice, possibly hampering the detection of small lesions. Variable sensitivity of different T1-weighted sequences to the T1-shortening effects of GBCA is another reason for variable detectability.

As contrast enhancement per se is unspecific for disease categorization related to disruption of the blood-brain barrier, time-resolved MRI techniques using GBCAs have been developed [21]. For example, MRI perfusion or perfusion-related techniques such as dynamic susceptibility contrast (DSC) and dynamic contrast-enhanced (DCE) MRI add specificity to contrast-enhanced MRI when assessing tumor grade and treatment-related changes. Non-contrast-enhanced MRI techniques that closely resemble these techniques are arterial spin labeling, time of flight, and phase-contrast MRI enabling perfusion estimation or vessel imaging.

## Useful non-contrast-enhanced MRI techniques

While contrast-enhancement is important to increase signal to background for small lesions, depict an impaired blood-brain barrier, accentuate vessel structures, and estimate tissue perfusion, new non-contrast-enhanced MRI techniques must be able to offer reliable alternatives to answer these clinical questions. There are useful sequences without GBCA, which shall be introduced here.

## Arterial spin labeling

Arterial spin labeling (ASL) is a non-contrast-enhanced technique that offers an estimation of brain perfusion such as cerebral blood flow (CBF) [22, 23]. Since the technique can depict tissue perfusion, it could replace GBCA-based MRI perfusion techniques such as dynamic susceptibility contrast (DSC) MRI and dynamic contrast-enhanced (DCE) MRI. In vessel imaging, ASL is often compared against digital subtraction angiography (DSA). With this technique, water protons (spins) in the blood are labeled magnetically by exposure to a radio-frequency pulse. These are then transported by the blood to the organ of interest and incorporated into the tissue by water exchange between blood and the tissue. Imaging is performed twice, once with (labeling acquisition) and once without the labeling (control acquisition). There are several variants of ASL pulse sequences. One variant is when the RF labeling is performed continuously (CASL) [24] and another where the labeling is applied on a large volume with one or two short RF pulses (PASL) [23]. A hybrid version of these two variants is the pseudocontinuous ASL (PCASL) where the continuous labeling pulse in CASL is replaced by a series of short pulses applied in the presence of a magnetic field gradient [22]. This method is currently recommended in the white paper by Alsop et al. [25]. For example, ASL has been tested for depicting arteriovenous malformations [26] and in the response assessment of cerebral tumors [27]. Main concerns with the technique include loss of signal due to susceptibility artifacts, motion artifacts, and low signal-to-noise ratio [28].

## Time of flight and phase-contrast MRI

Time of flight (TOF) MRI is a non-contrast-enhanced angiographic imaging method that measures and depicts the flow of blood inside a vessel compared to the surrounding static tissue [29]**.** TOF images depict vessel structures in 2D and 3D and can be used both for arteries and veins. Hence, it is a method that could be used instead of contrast-enhanced MR angiography (MRA). In TOF imaging, T1 hyperintense lesions such as lipomas can be mistaken for a vascular structure such as an aneurysm, although fat-saturated images and an awareness of artifacts can help distinguish these conditions [30]. Awareness should also be directed towards thrombus shine through in TOF MR arteriography and venography [31]. Source images as well as T1-weighted images should be scrutinized in order to differentiate between high T1 signal in thrombosed areas and normal flow [31].

Phase-contrast MRI is a non-contrast-enhanced sequence with a high rate of background suppression and excellent visualization of cerebral veins and high spatial resolution in 3D [32]. In phase-contrast MR venography, only moving tissue contributes to the MRI signal and static tissue gives no signal [32]. For example, TOF imaging can be of value when assessing for vessel patency in suspected occlusion or stenosis. Potential misinterpretations when assessing suspected dural sinus venous thrombosis can be related to sinus hypoplasia or atresia [31]. Interpretation also requires awareness of normal sinus filling defects such as arachnoid granulations and intrasinus septations [31].

## Diffusion MRI

Diffusion MRI or diffusion-weighted imaging (DWI) is a method that depicts the diffusion of protons in the tissue. In DWI, tissues with hindered or restricted proton movement will appear bright with a low apparent diffusion coefficient (ADC). DWI hence offers a depiction of the tissue that contrast-enhanced techniques cannot offer. More advanced diffusion-weighted techniques will allow visualizing the movement of protons along white matter tracts or estimation of perfusion metrics that could offer a substitution to contrast-enhanced perfusion MRI.

Changes in tissue microstructure due to pathological conditions can be quantified by diffusion MRI. In its most basic form, diffusion MRI yields maps of the apparent diffusion coefficient (ADC). In white matter, the ADC is anisotropic and depends on the diffusion-encoding direction due to the organized structure of membranes in and around axons [33]. This is the basis of diffusion tensor imaging and high angular resolution diffusion imaging (HARDI) methods for tractography [34]. Methods that go beyond ADC quantification and diffusion tensor imaging include diffusion kurtosis imaging (DKI) [35] and methods for microstructure imaging [36]. Such methods offer a more detailed characterization of the tissue. With encoding strategies that go beyond the standard diffusion MR method, more advanced techniques could become more specific for quantification of tissue properties such as cell size, cell count, cell membrane permeability, or cell shapes [36].

Diffusion MRI can also be used to acquire the perfusion fraction, which is related to the cerebral blood volume (CBV), by the so-called intravoxel incoherent motion imaging [37, 38]. Since perfusion imaging has shown to be a valuable tool in the medical investigations of, for example, stroke and tumors, intravoxel incoherent motion imaging is a potential diagnostic tool in these areas [39–42].

The main disadvantage of conventional DWI is that it is non-specific in terms of microstructure components, and observed values of the ADC can be affected by, for example, effects of flow apart from effects of diffusion [43]. Further, reading a DWI image necessitates a concurrent evaluation of ADC and/or T2-weighted images to assess potential T2 shine-through [43]. Finally, intravoxel incoherent motion (IVIM) perfusion estimates remain controversial and sensitive to echo time effects [44].

## Magnetic resonance spectroscopy

MR spectroscopy (MRS) provides information about the chemical composition of the tissue [45] and can be used for differential diagnosis and monitoring of treatment effects. It enables assessment of brain metabolism and can provide absolute metabolite concentrations, but in clinical practice, relative amounts (ratios) of different metabolites are usually reported [46]. MRS does not provide images of the brain, but rather a spectrum reflecting the chemical composition of the tissue in the selected volumes of interest. Currently, no contrast-enhanced MRI technique can assess tissue properties as does MRS. Useful areas for MRS are tumor characterization and treatment assessment.

Obstacles related to the introduction of MRS into clinical practice are related to the lack of standardization in terms of data acquisition and post-processing [47]. Other issues that also contribute to perceived difficulties with the technique include the placement of the MRS voxel, sensitivity for artifacts and motion, acquisition time, and post-processing of the data [47, 48].

## Susceptibility weighted imaging

Susceptibility weighted imaging (SWI) can be used to depict small areas in the brain causing inhomogeneity in the magnetic field rendering susceptibility artifacts. The possibility of depicting tiny structures with a high lesion-to-background signal is a potential advantage similar to that of contrast-enhanced T1-weighted images. Differences in magnetic susceptibility between deoxygenated and oxygenated blood render a phase difference between venous blood and surrounding tissue [49]. This difference is exploited in susceptibility weighted imaging to depict cerebral venous structures [49]. The method efficiently detects cerebral microbleeds, iron deposition, and cerebral calcifications [50].

Main disadvantages with this technique are the artifacts produced from subject motion or dental implants [51] as well as the sensitivity to blood oxygenation level [52].

## Amide proton transfer imaging

Amide proton transfer (APT) imaging was developed as a new contrast to assess the tissue pH and protein content by MRI [53]. Characterizing tissue properties based on pH and protein content could be an alternative to contrast-enhanced T1-weighted image depiction of impaired blood-brain barrier. The concept is based on the use of exchangeable protons to amplify the MR signal using a method called chemical exchange saturation transfer [54]. In a process similar to ASL, but now just inside the tissue, these exchangeable protons are first labeled using RF and transferred to water protons through physical exchange [55]. Fast repetition of this process leads to detection in MRI with sensitivity enhancements by factors of a hundred or more. This allows imaging of the signal from millimolar concentration of the exchangeable protons in these molecules with the molar sensitivity of water protons. Examples of groups containing these exchangeable protons are hydroxyls, amides, and amines. In APT-weighted (APTw) imaging, one uses the amide protons in mobile proteins and peptides in tissue as endogenous contrast. The signal intensity depends on the exchange rate between the amide and water protons and the number of amide protons. Therefore, two applications have been developed where one is sensitive to the change in exchange rate and the other depends on the protein concentration. One useful area for APT has been in brain tumor assessment. One disadvantage of APT in brain tumor imaging is the increased signal in proteinaceous cysts or hemorrhage that can be confounded with high-grade tumor if regular images are not carefully scrutinized for cystic or hemorrhagic tumor components [56]. Concerns have also been raised about the presence of proteins leaking from the vascular bed in the setting of a brain metastasis causing increased APT in the perifocal edema of brain metastasis [18].

## Specific clinical applications of non-contrast-enhanced MRI

### Cerebrovascular disease

#### Stroke

MRI is the most sensitive method to detect acute stroke [57] and is often used for cases that are challenging to diagnose or in follow-up. Acute stroke imaging aims to detect treatable causes of stroke and exclude mimickers. Imaging must rule out bleeding, identify thrombus, and differentiate non-salvageable tissue (infarct core) from the penumbra, but should also strive to assess the collateral circulation. DWI can aid in predicting outcome after stroke in posterior circulation stroke [58], and ASL-DWI mismatch has shown promise in identifying salvageable tissue in acute stroke [59] with an AUC of 0.76. ASL and DWI 24 h after stroke can be used to predict functional outcome in acute stroke patients with an AUC of 0.85 [60]. Further, susceptibility weighted imaging [61] or TOF [62] can be used to identify intraluminal thrombus or large vessel occlusion. However, a recent study shows that contrast-enhanced MRA shows higher accuracy (0.99) for detecting intracranial arterial occlusion compared to TOF (0.89) [63]. In summary, non-contrast-enhanced MRI seems to be able to replace contrast-enhanced techniques in clinical practice.

#### AVM and AV fistulas

Vessel imaging can be used for non-contrast-enhanced evaluation of AVM, AV fistulas, aneurysms, and steno-occlusive diseases including moyamoya. Imaging evaluates the disease processes before and after therapy and identifies complications after therapy. Imaging must be able to delineate the lesion and assess feeding and draining vessels. Perfusion maps from ASL combined with susceptibility weighted imaging (AUC = 0.97) have shown to be superior to conventional MRI (AUC = 0.93) and equal to digital subtraction angiography (DSA) in the preoperative assessment of AVM [64]. Further development of the ASL technique with super-selective ASL angiography shows a similar capacity as TOF to evaluate intracranial vessels and has shown promise in AVM evaluation [65].

Perfusion maps from ASL have shown equal diagnostic performance to DSA in the assessment of shunting in AVM [66] and to pre-therapeutically identify nidus, evaluating flow and AVM size reduction after therapy in a pediatric population [26]. Post-therapeutic evaluation of AVMs by ASL has also been described [67] with an AUC of 0.97. In a study evaluating ASL compared to DSA, results showed 100% sensitivity for ASL to evaluate AV-shunting and venous drainage in a pediatric population [68].

ASL angiography has shown high diagnostic performance for the evaluation of AV fistulas with excellent conformity between 4D ASL MR angiography and DSA in the identification of the fistula site and the venous drainage with an agreement of 100% [69], as quantified by the kappa value of 1.00 [70]. The kappa value is an index describing the agreement between two raters, and a complete agreement is described by a kappa value of 1. Perfusion maps from ASL are highly accurate in determining AVM Borden type and detect cortical venous drainage [71] with a sensitivity of 91% compared to DSA. Also, a 4D radial phase-contrast flow-tracking cartographic procedure showed good to excellent agreement between DSA and ASL [72] with kappa values of 0.92–1.00.

Aiming to improve contrast-enhanced MRA, time-resolved contrast-enhanced MR angiography has been developed and tested in patients with AV fistulas with accurate delineation of the fistula architecture in seven out of eight patients [73]. In summary, non-contrast-enhanced MRI seems to be able at least in part to replace contrast-enhanced techniques for vessel AV imaging.

#### Aneurysm

Pre-therapeutic imaging and post-therapeutic longitudinal follow-up must discern aneurysm size, location, and grade of occlusion. TOF MRA is the most widely used non-contrast-enhanced MR sequence for evaluation of cerebral aneurysms. Reports show that the performance of 3D TOF is equal to that of CT angiography [74] with an AUC of 0.91 and comparable to contrast-enhanced MRA [8] for assessing coiled aneurysms as occluded or patent. However, 3D TOF angiography showed more artifacts and lower detection rate of residual aneurysm patency compared with contrast-enhanced MR angiography [8]. Pre-therapeutic 3D TOF MRA was comparably accurate in detecting aneurysms as CT angiography with an AUC (alternative free-response ROC model) of 0.91 compared to DSA [74] and comparably good at describing aneurysm morphology. Interestingly, computer-aided design can help general radiologists to achieve a high aneurysm detection rate using 3D TOF MRA as shown by Hirai et al. in 2005 [75]. In summary, non-contrast-enhanced MRI seems to be able to replace contrast-enhanced MRI for untreated aneurysms but not for treated aneurysms.

#### Intracranial steno-occlusive carotid disease

Cerebral perfusion can be assessed using ASL as mentioned previously. Steno-occlusive carotid disease hinders the passage of blood proximal to the occlusion to enter a direct route to the brain. Imaging must be able to depict the cranial perfusion. The severity of symptoms is dependent on the existence of collateral circulation for example via the circle of Willis distal to the occlusion site and can be estimated by territorial ASL and TOF combined with comparable diagnostic quality to DSA [76] with kappa values of 0.70–0.72. 4D MRA ASL [77] or TOF alone [78] can estimate collateral flow in carotid artery steno-occlusive disease in a majority of patients and provide results comparable to DSA with intermodality agreement of kappa = 0.61 [77]. Since none of the included studies could show an excellent agreement between modalities, probably, non-contrast-enhanced MRI is not able to fully replace contrast-enhanced imaging for intracranial steno-occlusive carotid disease.

#### Moyamoya

Cerebral perfusion in non-atherosclerotic carotid stenosis (moyamoya disease) can be evaluated with ASL [79–82]. Specifically, ASL angiography can visualize cerebral vessels and moyamoya vessels in non-operated hemispheres and stage moyamoya with an accuracy of 86–100% compared to DSA [79]. ASL can identify changes in CBF in patients with moyamoya disease treated after revascularization (kappa = 0.77 for collateral grading compared with DSA), but does not allow for reliable anastomosis patency (kappa = 0.57), which is better appreciated by DSA [82]. In summary, non-contrast MRI seems to be able to replace contrast-enhanced MRI in the pre-therapeutic stage but not postoperatively.

#### Vasculitis

Imaging in patients with vasculitis must be able to depict direct signs of vasculitis in the affected vessel as well as tissue-based complications of vasculitis such as hemorrhage and stroke. Although contrast-enhanced MRA can show direct signs of cerebral artery vasculitis by contrast enhancement in the vessel wall, notably the American College of Radiology Appropriateness Criteria for cerebrovascular disease show the same diagnostic rating for MRA head without GBCA as for head MRA with GBCA [83]. Importantly, DSA is still considered the imaging gold standard, and hence, non-contrast MRI seems to only partly be able to replace contrast-enhanced techniques.

#### Cerebral venous thrombosis

Imaging in cerebral venous thrombosis must be able to identify the clot and the lack of flow in the affected venous structure as well as potential parenchymal effects of venous stasis. The clinical presentation of CVT is variable, and thus, pre-imaging clinical suspicion of cerebral venous thrombosis is difficult [84]. MRI can detect secondary ischemic and hemorrhagic areas as well as localizing and describing the extent of the thrombosis without the use of GBCA through 3DT1 turbo spin echo (sensitivity and specificity 97–100%) and 2D-TOF (sensitivity and specificity 85–93%) [85]. Previous data show that contrast-enhanced imaging (AUC = 0.99) is superior to non-contrast-enhanced 2D-TOF MR venography (AUC = 0.88–0.89) to detect cerebral venous thrombosis [9]. This has also been confirmed by one more recent study [86]. However, in a recent study evaluating non-contrast-enhanced MRI techniques, 3D phase-contrast MR venography shows high diagnostic accuracy (sensitivity 100%, specificity 71%), especially when combining with non-contrast-enhanced CT and conventional non-contrast-enhanced MRI [87]. Non-contrast-enhanced MRI seems to be able to partly replace contrast-enhanced techniques in cerebral venous thrombosis, especially when combining with non-contrast-enhanced CT.

Table 1 summarizes the diagnostic performance in the studies described above.

**Table 1:** Diagnostic accuracy measures in non-contrast-enhanced MRI techniques and contrast-enhanced techniques in cerebrovascular disease

Clinical question	Diagnostic performance non-CE	Diagnostic performance CE gold standard or DSA gold standard	Author (year)
Detect cerebral venous thrombosis	AUC 0.89 (± 0.03 SD) 2D-TOF MR venography	AUC 0.99 CE T1 3D MP-RAGE	Liang et al. (2001)
Detect cerebral venous thrombosis	80% sensitivity, 65% specificity	CE MRV reference standard	Bernard (2017)
Detect cerebral venous thrombosis	Accuracy 92.7% conv non-contrast-enhanced sequences	Accuracy 98.3 CE T1 3D GRE	Sari (2015)
Detect cerebral venous thrombosis	Sensitivity/specificity100%/71% 3D PC-MR venography	DSA gold standard	Ozturk et al. (2018)
Detect intracranial arteriovenous shunting in AVM	AUC 0.97 (95% CI 0.90–1.00) CBF ASL/SWI	AUC 0.93 (95% CI 0.87–0.97) conv MRI including CE T1 and CE MRA, DSA reference standard	Hodel et al. (2017)
Nidus localization in AVM	Sensitivity 100% CBF ASL	DSA gold standard	Blauwblomme et al. (2015)
Evaluation of AVM obliteration	AUC 0.94 CBF ASL	DSA gold standard	Kodera et al. (2017)
Detect arteriovenous shunting and venous drainage in children with AVM	Sensitivity 100% CBF ASL	DSA gold standard	Nabavizadeh et al. (2014)
Identify fistula site and venous drainage in AV fistula	Kappa 1.00 four-dimensional MR angiography ASL	DSA gold standard	Iryo et al. (2014)
Detect and localize AV fistula	Sensitivity 91% (95% CI 69–98) CBF ASL	DSA gold standard	Amukotuwa et al. (2016)
Characterize dural AV fistula: define shunt location/feeding artery/draining vein/Cognard classification	Kappa interreader agreement 1.00/0.92/1.00/1.00 flow-tracking cartography	DSA gold standard	Edjlali et al. (2014)
Detect intracranial aneurysms	AUC 0.91 TOF MRA	AUC 0.91 CT angiography/DSA gold standard	Hiratsuka et al. (2008)
Moyamoya Suzuji stage	Accuracy > 86 (0.86–1 range) ASL-4D MRA	DSA gold standard	Uchino et al. (2015)
Predictor of 24-h DWI lesion in non-reperfused ischemic stroke	AUC 0.76 (95% CI 0.63–0.85) CBF ASL	AUC 0.79 (95% CI 73–84) Tmax DSC	Bivard et al. (2014)
Detect arterial occlusion in stroke	Accuracy TOF MRA 0.89	Accuracy CE MRA 0.99	Dhundass et al. (2019)

*ASL* arterial spin labeling, *AUC* area under curve, *AV* arteriovenous, *AVM* arteriovenous malformation, *CBF* cerebral blood flow, *CE* contrast-enhanced, *conv* conventional, *CT* computed tomography, *DSA* digital subtraction angiography, *DSC* dynamic susceptibility weighted, *DWI* diffusion-weighted imaging, *MRA* magnetic resonance angiography, *MRV* magnetic resonance venography, *PC* phase contrast, *SWI* susceptibility weighted imaging, *TOF* time of flight

### Multiple sclerosis

Multiple sclerosis is the classical indication to use GBCA in neuroimaging [88, 89]. Contrast-enhanced MRI decreases time to diagnosis and helps to identify and characterize multiple sclerosis mimics at first clinical presentation [90]. However, the presumption that all patients with multiple sclerosis should undergo contrast-enhanced MRI has started to change recently. Follow-up imaging of definite disease does not per se require GBCA administration, although GBCA can be used to re-evaluate the original diagnosis or as new baseline before therapeutic changes [91]. Previous studies showed a modest correlation between contrast enhancement and clinical outcome [92]. For example, the relapse rate is not influenced by MRI enhancement status when taking other covariates into account such as disease duration [92] and the relapse is not predicted by the presence of gadolinium-enhancing lesions on MRI [93].

Furthermore, several studies have been able to predict contrast enhancement by analysis of non-contrast-enhanced T1- and T2-weighted images MRI [94, 95] with an AUC of 0.72–0.83, by quantification of fractional anisotropy from diffusion tensor imaging [96] with an AUC of 0.93, and by texture analysis of T2-weighted images [97]. Magnetization transfer ratio quantification can also differentiate between contrast-enhancing and non-contrast-enhancing lesions in patients with multiple sclerosis, likely representing the affection of the disease on the BBB integrity [98].

Non-contrast-enhanced MRI of T2 lesion load and cerebral atrophy show a strong correlation (*R*^2^ = 0.74) with clinical status [99], and T2 lesion volume only has a moderate correlation with clinical disability at long term follow-up [100]. Newer volumetric quantitative techniques have shown promise in assessing radiological disease status [101], but they still lack clear correlation to the clinical status of the patients. It can also be noted that patients presenting with multiple sclerosis are relatively young and subject to repeated examinations, which strengthens the case for reducing the use of GBCA in this patient group. Non-contrast-enhanced MRI seems to be able to replace contrast-enhanced MRI for longitudinal follow-up of patients but not at first presentation. Table 2 summarizes the diagnostic performance in the studies described above.

**Table 2:** Diagnostic accuracy measures in non-contrast-enhanced MRI techniques and contrast-enhanced techniques in multiple sclerosis

Clinical question	Diagnostic performance non-CE	Diagnostic performance CE gold standard	Author (year)
Predict contrast enhancement in multiple sclerosis	AUC 0.83 (95% CI 0.80–0.87) non-enhanced conv MRI and logistic regression model fitting	CE T1 reference standard	Shinohara et al. (2012)
Predict contrast enhancement in multiple sclerosis	AUC 0.72 T2 burden of disease	CE T1 reference standard	Barkhof et al. (2005)
Predict contrast enhancement in multiple sclerosis	AUC 0.93 (95% CI 0.87–0.99) T2W, SDC, QSM	CE T1 reference standard	Gupta et al. (2018)
Predict contrast enhancement in multiple sclerosis	T2-weighted texture parameters 86% sensitivity, 84% specificity	CE T1 reference standard	Michoux et al. (2015)

*AUC* area under curve, CE contrast-enhanced, *conv* conventional, *GBCA* gadolinium-based contrast agent, *SDC* statistical detection of change, *QSM* quantitative susceptibility mapping

### Primary and secondary brain tumor

#### Tumor extent and detection

Brain tumor detection and characterization is an area where contrast-enhanced MRI is frequently used [102, 103]. However, since these first report on the application of contrast-enhanced MRI, techniques for non-contrast-enhanced MRI have evolved. As tumor localization and extent are two essential issues in pre-operative brain tumor imaging, non-contrast-enhanced MRI should be able to detect and localize primary brain tumors. This can be achieved by for example T2 FLAIR imaging showing high signal intensity in low-grade gliomas [104]. In high-grade gliomas, increased signal on T2 FLAIR can encompass tumor edema with tumor cells [105]. Imaging-histology correlation data show that primary malignant brain tumors rarely have surrounding edema without interspersed tumor cells outside the contrast-enhanced area [20]. Compared to contrast-enhanced MRI, diffusion imaging can better depict the perifocal tumor density and effects on surrounding white matter tracts [106, 107]. Further issues in brain tumor imaging relate to the characterization of tumor type and malignancy grade. It is known that the contrast enhancement is an imperfect marker of malignancy grade [15]. In comparison to primary brain tumors, cerebral metastases can be multiple and small with potential increased lesion detection rate by the use of GBCAs [108].

Although it may be feasible to exclude the use of GBCA without limiting the ability to detect primary brain tumors, imaging of secondary brain tumors cannot be performed without GBCA. One study reported that 20% of cerebral metastasis were undetected on pre-contrast echo-planar imaging FLAIR [109]. The use of contrast-enhanced T2 FLAIR has shown higher sensitivity (100%) compared with early phase 3DT1 GRE (76%) [110]. Contrast-enhanced MRI is compulsory for the detection of small metastases. However, even though GBCA is used (contrast-enhanced T1), leptomeningeal metastasis confirmed by cerebrospinal fluid analysis may not be detected [111]. In summary, non-contrast-enhanced MRI seems not to be able to replace contrast-enhanced MRI in patients with metastatic disease.

#### Differentiation of brain tumors

A few recent studies demonstrated that support vector machine-based classifiers using histogram features of the ADC from diffusion MRI (AUC = 0.97) could better discriminate between different types of posterior fossa tumors in children than contrast-enhanced T1 or T2 images (AUC = 0.84) [112]. Texture features from conventional non-contrast-enhanced MRI showed sufficient accuracy to separate childhood tumors into the correct classes (accuracy 71–92%), which is higher than the clinical radiological reading of the cases (47% correct diagnosis) [113].

Non-contrast-enhanced MRI can also differentiate between cerebral lymphoma and GBM by the use of ADC [114] with an AUC of 0.94. This can also be achieved by ASL [115] with an AUC of 0.91 and an accuracy of 95%. MRS can discriminate between metastases and CNS lymphoma/GBM with equal diagnostic performance (AUC = 0.96) as perfusion MRI with contrast agent injection (AUC = 0.97) [116]. Further, ASL [117], T2 relaxometry [118], DKI [119], and APTw imaging [18] can discriminate between cerebral metastasis and GBM with sufficient diagnostic performance (AUC = 0.84, 0.86, 1.00, and 0.91, respectively). Aiming to increase the specificity of contrast-enhanced imaging, one can use perfusion weighting such as dynamic susceptibility contrast MRI. This will allow discrimination between glioblastoma, metastasis, and primary central nervous system lymphoma with accuracy between 0.94 and 0.99 [120].

#### Tumor grade discrimination

High-grade primary brain tumors are not always contrast enhancing. In fact, up to 18% of high-grade gliomas can be non-enhancing [15]. Magnetic resonance spectroscopy (MRS) (AUC 0.90) and APTw (AUC = 0.82) have shown higher diagnostic performance to grade brain tumors in low- or high-grade, than conventional MRI including contrast-enhanced T1-weighted images (AUC = 0.65) [121]. One meta-analysis pooling 83 articles showed the value of using Cho/Cr and Cho/NAA as well as contrast-enhanced dynamic perfusion-weighted imaging to differentiate between high-grade and low-grade gliomas [122]. APTw imaging has shown comparable discriminatory potential to DSC MRI between low- and high-grade brain tumors [123] with an AUC of 0.85–0.86 for ASL compared to 0.80–0.82 for DSC. Further, both ASL [10], DKI [124], and intravoxel incoherent motion imaging [125] can discriminate between low- and high-grade gliomas (AUC = 0.93-0.96).

#### Follow-up after treatment

In the follow-up of patients treated with surgery and/or radiochemotherapy for brain tumor, the updated Response Assessment in Neuro-Oncology criteria (RANO) consider not only contrast enhancement but also progression of T2 FLAIR changes [17, 126].

Pseudoprogression and pseudoresponse are two entities that are challenging for the neuroradiologist when evaluating post-therapeutic intracranial tumors on contrast-enhanced MRI [17, 126]. Non-contrast-enhanced MRI that has shown promise in depicting and discriminating between treatment-related changes and tumor progression includes ASL (AUC = 0.84) [27], APTw [127, 128], intravoxel incoherent motion imaging (AUC = 0.94-0.95) [37], diffusion tensor imaging (AUC = 0.84) [129], and MRS (AUC = 0.91) [130]. One recent study showed how quantitative T1 mapping (without GBCA) could be used to monitor GBM during bevacizumab treatment [131]. Another study utilizing non-contrast-enhanced texture analysis showed high diagnostic performance to differentiate between recurrent tumor and treatment-related changes [132] with an AUC of 0.79 compared to 0.57 for contrast-enhanced T1-weighted imaging. MRS has shown promise in discriminating between tumor recurrence and treatment-related changes by measuring elevated choline [133–136]. More surprisingly, in patients with diffuse intrinsic pontine glioma, ASL post-radiotherapy shows high CBF in patients with pseudoprogression [137]. Identifying signs of pseudoprogression is of importance since the misinterpretation of contrast-enhancement as a progressive disease might lead to discontinuation of therapy [17]. Notably, the use of GBCA in the follow-up of initially non-contrast-enhanced tumors or tumors that lack known potential to malignify can be questioned. The specificity of contrast-enhanced imaging can be increased by perfusion weighting such as dynamic susceptibility contrast MRI. A meta-analysis from 2018 showed a sensitivity of 82% and a specificity of 95% to discriminate between true progression and pseudoprogression by DSC [138]. In summary, non-contrast-enhanced MRI seems to at least in part be able to replace contrast-enhanced MRI techniques in the diagnosis and follow-up of primary brain tumors.

Table 3 summarizes the above-described diagnostic performance.

**Table 3:** Diagnostic accuracy measures in non-contrast-enhanced MRI techniques and contrast-enhanced techniques in brain tumor imaging

Clinical question	Diagnostic performance non-CE	Diagnostic performance CE gold standard	Author (year)
Astrocytic tumor grading	AUC 0.96 (95% CI 0.84–1.0) CBF ASL	AUC 0.98 (95% CI 0.87–1.00) CBF DSC	Morana et al. (2018)
Glioma grading	AUC 0.82 (95% CI 0.62–1.00) APTw mean, AUC 0.90 (95% CI 0.73–1.00) Cho/Cr MRS	AUC 0.65 (0.47–0.84) CE T1	Sakata et al. (2017)
Glioma grading	AUC 0.85–0.86 (95% CI 0.74–0.92 and 95% CI 0.75–0.94) APTw90	AUC 0.80–0.82 (95% CI 0.64–0.89 and 0.67–0.90) nCBV90 DSC	Park et al. (2015)
Pediatric posterior fossa grading	AUC 0.97 ADC (classification rate) DWI	AUC 0.84 CE T1 (classification rate)	Rodriguéz Gutierrez et al. (2014)
Discriminate between CNS lymphoma and GBM	AUC 0.94 ADC DWI	Equal rate of CE T1 contrast enhancement between groups	Ko et al. (2016)
Discriminate between CNS lymphoma and GBM	Accuracy 0.91 (95% CI 0.84–0.95) CBF ASL	Accuracy 93–95% conv MRI including CE T1	You et al. (2018)
Discriminate between metastases and CNS lymphoma/GBM	AUC 0.96 Lac/Cr MRS	AUC 0.97 PSRmax DSC	Vallée et al. (2018)
Progression vs pseudoprogression in GBM	AUC 0.84 (95% CI 0.72–0.96) linear anisotropy DTI	AUC 0.77 (95% CI 0.63–0.92) rCBVmax DSC	Wang et al. (2016)
Progression vs pseudoprogression in glial tumors and brain metastases	AUC 0.79 (95% CI 0.77–0.81) T2FLAIR	AUC 0.57 (± 0.08) CE T1	Tiwari et al. (2016)
Progression vs pseudoprogression in glioma	AUC 0.82 CBF ASL	AUC 0.84 nrCBV DSC	Wang et al. (2018)
Progression vs pseudoprogression in metastases	AUC 0.94-0.95 (95% CI 0.87–0.98 and 0.88-0.98) IVIM	AUC 0.91–0.93 (95% CI 0.83–0.96 and 0.86–0.98) DSC + DWI	Kim et al. (2014)
Progression vs pseudoprogression in GBM	AUC 0.89 APTw90	AUC 0.77 and 0.80 CBV DSC	Park et al. (2016)
Detection of cerebral metastasis	Sensitivity 0.80% FLAIR-EPI	Sensitivity 100% SE-T1W	Tomura et al. (2007)

*ADC* apparent diffusion coefficient, *ASL* arterial spin labeling, *AUC* area under curve, *CBF* cerebral blood flow, *CE* contrast-enhanced, *CNS* central nervous system, *conv* conventional, *DSC* dynamic susceptibility weighted, *DTI* diffusion tensor imaging, *DWI* diffusion-weighted imaging, *FLAIR* fluid attenuated inversion recovery, *GBM* glioblastoma, *IVIM* intravoxel incoherent motion, *MRS* magnetic resonance spectroscopy, *nrCBV* normalized relative cerebral blood volume, *PSR* percentage of signal recovery, *rCBVmax* maximum relative cerebral blood volume

### Cerebral abscess and infectious meningitis

Intracranial and cerebral infections are classical indications for contrast-enhanced MRI. However, abscesses and necrotic tumors can have the same appearance and present as ring-enhancing lesions on contrast-enhanced T1-weighted images [139]. Hence, the exclusive use of contrast-enhanced T1 sequences or contrast-enhanced CT will not help in determining the etiology of the lesion. However, bacterial cerebral abscess can be differentiated from a necrotic or cystic brain tumor by DWI (AUC = 0.96) [140] with higher diagnostic performance than morphological T1 and T2 images; this has also been reported by other groups [139] (AUC for DWI = 1.00) as shown in Table 4. DWI has replaced MRS as the method of choice for differentiating between abscesses and necrotic tumors such as glioblastomas and metastases. However, the use of DWI in more uncommon non-pyogenic abscesses is less valid and unable to correctly classify the lesion.

**Table 4:** Diagnostic accuracy measures in non-contrast-enhanced MRI techniques and contrast-enhanced techniques in brain infection

Clinical question	Diagnostic performance non-CE	Diagnostic performance CE gold standard	Author (year)
Abscess detection	Specificity 100% ADC	CE T1 and T2 signal intensity could not distinguish between groups	Nadal et al. (2003)
Detecting infectious meningitis	Sensitivity 33% T2 FLAIR	Sensitivity 100% T2 FLAIR	Splendiani et al. (2005)
Detecting infectious meningitis	Sensitivity 25% 3DT2FLAIR	Sensitivity 75% CE 3DT2FLAIR	Fukuoka et al. (2010)

*ADC* apparent diffusion coefficient, *AUC* area under curve, *CE* contrast-enhanced, *FLAIR* fluid attenuated inversion recovery

Although historically not considered a radiological diagnosis, detection of meningitis has shown higher sensitivity on contrast-enhanced T2 FLAIR images (100% sensitivity) compared to non-contrast-enhanced T2 FLAIR (33% sensitivity) or contrast-enhanced T1 (50% sensitivity) [141]. Another study showed a higher sensitivity to detect meningitis by contrast-enhanced T2 FLAIR (75% sensitivity) compared to non-contrast-enhanced 3DT2FLAIR (25% sensitivity) [142]. In ventricular infection, DWI can assess the presence of intraventricular pus [86, 143]. Except for meningitis, non-contrast-enhanced MRI using DWI seems to be able to replace contrast-enhanced MRI for the detection of cerebral abscess. In fact, for abscess detection, DWI seems superior to contrast-enhanced techniques.

## Discussion

In this review, we have summarized data on how non-contrast-enhanced MRI can detect and characterize pathology in, at least, ten specific diseases with a diagnostic performance comparable with contrast-enhanced MRI for specific clinical questions. Although we agree that GBCA can increase lesion detection (sensitivity), the specificity in lesion characterization by contrast-enhanced MRI is often low compared to other more advanced MR sequences. In fact, the high sensitivity and the low specificity can mislead clinical decisions [15–20]. Contrast agent injection—to achieve a high lesion-to-background contrast—is relatively less important in MRI compared to CT with inherent much higher soft tissue contrast.

In general, the data summarized in this review showed the lowest diagnostic performance for static GBCA MRI such as contrast-enhanced T1 but higher performance for dynamic GBCA MRI such as DSC perfusion. This highlights the importance of depicting physiological properties of GBCA injection such as perfusion of the tissue to increase diagnostic accuracy in clinical practice.

Although new clinical guidelines [5, 144–148] increase the awareness of GBCA usage, we believe there is still a long way to go before GBCA can be fully replaced by other imaging techniques. In fact, there are many diseases and situations where an MRI scan cannot be reliably assessed based on non-contrast-enhanced imaging alone. Examples based on this review are detection of meningitis, cerebral metastasis, and dural sinus venous thrombosis. The main reason to use GBCA is to increase the sensitivity in detecting a disease, since a missed disease can lead to the wrong or no treatment. Furthermore, this review predominately included articles focused on specific sequences and not full clinical protocols. Even though specific non-contrast-enhanced sequences were found to have the capacity to aid clinical diagnosis, in reality, a routine clinical MRI protocol includes several imaging sequences and contrast-enhanced imaging is still one important part. We also have to be aware that first reports on new techniques are often more positive than later published studies [149] and that these new techniques have downsides such as prolonged scan time, motion sensitivity, and lack of availability. In patients with short estimated survival, the potential long-term risk of GBCA can be overlooked. The use of GBCA might be more important in primary brain imaging at first clinical presentation and less needed for follow-up scanning especially when dealing with children that get repeated scanning at young age or patients with chronic diseases like multiple sclerosis. Clinical examples where non-contrast-enhanced MRI can be used instead of contrast-enhanced MRI already today are in the evaluation of, for example, dural sinus venous thrombosis in pregnant women [150]. Other clinical examples include patients with intracranial pathology that also have kidney failure or in premature infants.

This review highlights that non-contrast-enhanced MRI techniques can be used in several diseases affecting the brain, but many diseases have not been covered in this review. In addition, there are few published studies focusing on the comparison of non-contrast-enhanced and contrast-enhanced MRI and there is still a lack of evidence to include or exclude GBCAs from clinical routine protocols for many diseases.

We suggest that to further evaluate the use of GBCA in routine clinical protocols for different brain disease, single or multicenter studies should be performed evaluating a random sample of neuroimaging cases investigated with both non-contrast-enhanced and contrast-enhanced MRI. Multiple independent reviewers should be used to further clarify when GBCA is most valuable with regard to the level of clinical confidence for diagnosing and characterizing lesions.

Further, the cost benefit and patient benefit must be considered when deciding on protocol optimization as well as the potential associated risks. There are several advantages when not injecting GBCA, for example, lower the indication for an intravenous line which facilitates the logistics for the patients with no need for serum creatinine blood sample before MRI scanning, reduced cost of contrast media, reduced preparation time for scanning, and reduced risk of adverse events.

One last caveat is how imaging really affects the outcome of the patients. In general, there is a shortage of radiological studies validating their findings towards defined clinical endpoints such as symptoms or survival. Further studies should direct the attention towards the added benefit for patient outcome using GBCAs.

## Conclusion

This review presents non-contrast-enhanced alternatives in MR neuroimaging for ten specific diseases and describes the advantages and disadvantages of ASL, TOF, phase contrast, DWI, MRS, SWI, and APT imaging together with data on diagnostic performance compared to contrast-enhanced alternatives.

## Data Availability

Not applicable

## Abbreviations

ADC: Apparent diffusion coefficient
APTw: Amide proton transfer-weighted
ASL: Arterial spin labeling
AV: Arteriovenous
AVM: Arteriovenous malformation
CBF: Cerebral blood flow
DCE: Dynamic contrast-enhanced
DKI: Diffusion kurtosis imaging
DSA: Digital subtraction angiography
DSC: Dynamic susceptibility contrast
GBCA: Gadolinium-based contrast agent
GBM: Glioblastoma
IVIM: Intravoxel incoherent motion
MRA: Magnetic resonance angiography
MRS: Magnetic resonance spectroscopy
PC: Phase contrast
RF: Radio-frequency
TOF: Time of flight

## References

[1] Runge VM, Clanton JA, Price AC, Wehr CJ, Herzer WA, Partain CL (1985). The use of Gd DTPA as a perfusion agent and marker of blood-brain barrier disruption. Magn Reson Imaging..

[2] EMA (2017) EMA’s final opinion confirms restrictions on use of linear gadolinium agents in body scans. Available via www.ema.europa.eu/documents/press-release/emas-final-opinion-confirmsrestrictions-use-linear-gadolinium-agents-body-scans_en.pdf. Accessed on 30 Nov 2018.

[3] Wang Y, Alkasab TK, Narin O, Nazarian RM, Kaewlai R, Kay J (2011). Incidence of nephrogenic systemic fibrosis after adoption of restrictive gadolinium-based contrast agent guidelines. Radiology.

[4] Bennett CL, Qureshi ZP, Sartor AO, Norris LB, Murday A, Xirasagar S (2012). Gadolinium-induced nephrogenic systemic fibrosis: the rise and fall of an iatrogenic disease. Clin Kidney J.

[5] Gulani V, Calamante F, Shellock FG, Kanal E, Reeder SB (2017). Gadolinium deposition in the brain: summary of evidence and recommendations. Lancet Neurol..

[6] McDonald RJ, McDonald JS, Kallmes DF, Jentoft ME, Paolini MA, Murray DL (2017). Gadolinium deposition in human brain tissues after contrast-enhanced mr imaging in adult patients without intracranial abnormalities. Radiology.

[7] Lord ML, Chettle DR, Gräfe JL, Noseworthy MD, McNeill FE (2018). Observed deposition of gadolinium in bone using a new noninvasive in vivo biomedical device: results of a small pilot feasibility study. Radiology.

[8] Anzalone N, Scomazzoni F, Cirillo M, Righi C, Simionato F, Cadioli M (2008). Follow-up of coiled cerebral aneurysms at 3T: comparison of 3D time-of-flight MR angiography and contrast-enhanced MR angiography. AJNR Am J Neuroradiol.

[9] Liang L, Korogi Y, Sugahara T, Onomichi M, Shigematsu Y, Yang D (2001). Evaluation of the intracranial dural sinuses with a 3D contrast-enhanced MP-RAGE sequence: prospective comparison with 2D-TOF MR venography and digital subtraction angiography. AJNR Am J Neuroradiol.

[10] Morana G, Tortora D, Staglianò S, Nozza P, Mascelli S, Severino M (2018). Pediatric astrocytic tumor grading: comparison between arterial spin labeling and dynamic susceptibility contrast MRI perfusion. Neuroradiology.

[11] Smith APL, Marino M, Roberts J, Crowder JM, Castle J, Lowery L (2017). Clearance of gadolinium from the brain with no pathologic effect after repeated administration of gadodiamide in healthy rats: an analytical and histologic study. Radiology.

[12] Zhao Z, Nelson AR, Betsholtz C, Zlokovic BV (2015). Establishment and dysfunction of the blood-brain barrier. Cell.

[13] Obermeier B, Daneman R, Ransohoff RM (2013). Development, maintenance and disruption of the blood-brain barrier. Nat Med..

[14] Louis DN, Perry A, Reifenberger G, von Deimling A, Figarella-Branger D, Cavenee WK (2016). The 2016 World Health Organization Classification of Tumors of the Central Nervous System: a summary. Acta Neuropathol.

[15] Scott JN, Brasher PMA, Sevick RJ, Rewcastle NB, Forsyth PA (2002). How often are nonenhancing supratentorial gliomas malignant? A population study. Neurology.

[16] Stockham AL, Tievsky AL, Koyfman SA, Reddy CA, Suh JH, Vogelbaum MA (2012). Conventional MRI does not reliably distinguish radiation necrosis from tumor recurrence after stereotactic radiosurgery. J Neurooncol.

[17] Wen PY, Macdonald DR, Reardon DA, Cloughesy TF, Gregory Sorensen A, Galanis E (2010). Updated response assessment criteria for high-grade gliomas: response assessment in neuro-oncology working group. J Clin Oncol.

[18] Yu H, Lou H, Zou T, Wang X, Jiang S, Huang Z (2017). Applying protein-based amide proton transfer MR imaging to distinguish solitary brain metastases from glioblastoma. Eur Radiol..

[19] Lev MH, Ozsunar Y, Henson JW et al (2014). Glial tumor grading and outcome prediction using dynamic spin-echo MR susceptibility mapping compared with conventional contrast-enhanced MR: confounding effect of elevated rCBV of oligodendrogliomas [corrected]. AJNR Am J Neuroradiol. 2004;25: 214–221. PMC7974605 14970020

[20] Zetterling M, Roodakker KR, Berntsson SG, Edqvist P-H, Latini F, Landtblom A-M (2016). Extension of diffuse low-grade gliomas beyond radiological borders as shown by the coregistration of histopathological and magnetic resonance imaging data. J Neurosurg..

[21] Rosen Bruce R., Belliveau John W., Vevea James M., Brady Thomas J. (1990). Perfusion imaging with NMR contrast agents. Magnetic Resonance in Medicine.

[22] Dai W, Garcia D, de Bazelaire C, Alsop DC (2008). Continuous flow-driven inversion for arterial spin labeling using pulsed radio frequency and gradient fields. Magn Reson Med..

[23] Edelman RR, Siewert B, Adamis M, Gaa J, Laub G, Wielopolski P (1994). Signal targeting with alternating radiofrequency (STAR) sequences: application to MR angiography. Magn Reson Med..

[24] Detre JA, Alsop DC, Vives LR, Maccotta L, Teener JW, Raps EC (1998). Noninvasive MRI evaluation of cerebral blood flow in cerebrovascular disease. Neurology..

[25] Alsop DC, Detre JA, Golay X, Günther M, Hendrikse J, Hernandez-Garcia L (2015). Recommended implementation of arterial spin-labeled perfusion MRI for clinical applications: a consensus of the ISMRM perfusion study group and the European consortium for ASL in dementia. Magn Reson Med..

[26] Blauwblomme T, Naggara O, Brunelle F, Grévent D, Puget S, Di Rocco F (2015). Arterial spin labeling magnetic resonance imaging: toward noninvasive diagnosis and follow-up of pediatric brain arteriovenous malformations. J Neurosurg Pediatr..

[27] Wang Y-L, Chen S, Xiao H-F, Li Y, Wang Y, Liu G (2018). Differentiation between radiation-induced brain injury and glioma recurrence using 3D pCASL and dynamic susceptibility contrast-enhanced perfusion-weighted imaging. Radiother Oncol..

[28] Deibler AR, Pollock JM, Kraft RA, Tan H, Burdette JH, Maldjian JA (2008). Arterial spin-labeling in routine clinical practice, part 3: hyperperfusion patterns. AJNR Am J Neuroradiol..

[29] Nishimura Dwight G., Macovski Albert, Pauly John M., Conolly Steven M. (1987). MR angiography by selective inversion recovery. Magnetic Resonance in Medicine.

[30] Kemmling A, Noelte I, Gerigk L, Singer S, Groden C, Scharf J (2008). A diagnostic pitfall for intracranial aneurysms in time-of-flight MR angiography: small intracranial lipomas. AJR Am J Roentgenol..

[31] Leach JL, Fortuna RB, Jones BV, Gaskill-Shipley MF (2006). Imaging of cerebral venous thrombosis: current techniques, spectrum of findings, and diagnostic pitfalls. Radiographics.

[32] Pernicone JR, Siebert JE, Potchen EJ, Pera A, Dumoulin CL, Souza SP (1990). Three-dimensional phase-contrast MR angiography in the head and neck: preliminary report. AJR Am J Roentgenol..

[33] Beaulieu Christian (2002). The basis of anisotropic water diffusion in the nervous system - a technical review. NMR in Biomedicine.

[34] Hagmann P, Jonasson L, Deffieux T, Meuli R, Thiran J-P, Wedeen VJ (2006). Fibertract segmentation in position orientation space from high angular resolution diffusion MRI. Neuroimage..

[35] Liu C, Bammer R, Acar B, Moseley ME (2004). Characterizing non-Gaussian diffusion by using generalized diffusion tensors. Magn Reson Med..

[36] Nilsson Markus, Englund Elisabet, Szczepankiewicz Filip, van Westen Danielle, Sundgren Pia C. (2018). Imaging brain tumour microstructure. NeuroImage.

[37] Kim DY, Kim HS, Goh MJ, Choi CG, Kim SJ (2014). Utility of intravoxel incoherent motion MR imaging for distinguishing recurrent metastatic tumor from treatment effect following gamma knife radiosurgery: initial experience. AJNR Am J Neuroradiol.

[38] Le Bihan D, Breton E, Lallemand D, Aubin ML, Vignaud J, Laval-Jeantet M (1988). Separation of diffusion and perfusion in intravoxel incoherent motion MR imaging. Radiology..

[39] Heit JJ, Wintermark M, Martin BW, Zhu G, Marks MP, Zaharchuk G (2018). Reduced intravoxel incoherent motion microvascular perfusion predicts delayed cerebral ischemia and vasospasm after aneurysm rupture. Stroke..

[40] Gao Qian-Qian, Lu Shan-Shan, Xu Xiao-Quan, Wu Cheng-Jiang, Liu Xing-Long, Liu Sheng, Shi Hai-Bin (2016). Quantitative assessment of hyperacute cerebral infarction with intravoxel incoherent motion MR imaging: Initial experience in a canine stroke model. Journal of Magnetic Resonance Imaging.

[41] Yamashita K, Hiwatashi A, Togao O, Kikuchi K, Kitamura Y, Mizoguchi M (2016). Diagnostic utility of intravoxel incoherent motion MR imaging in differentiating primary central nervous system lymphoma from glioblastoma multiforme. J Magn Reson Imaging..

[42] Le Bihan D, Breton E, Lallemand D, Grenier P, Cabanis E, Laval-Jeantet M (1986). MR imaging of intravoxel incoherent motions: application to diffusion and perfusion in neurologic disorders. Radiology..

[43] Roberts TPL, Rowley HA (2003). Diffusion weighted magnetic resonance imaging in stroke. Eur J Radiol..

[44] Feng Z, Min X, Wang L, Yan X, Li B, Ke Z (2018). Effects of echo time on IVIM quantification of the normal prostate. Sci Rep..

[45] Bottomley PA, Hart HR, Edelstein WA, Schenck JF, Smith LS, Leue WM (1983). NMR imaging/spectroscopy system to study both anatomy and metabolism. Lancet..

[46] Burtscher IM, Holtås S (2001). Proton MR spectroscopy in clinical routine. J Magn Reson Imaging..

[47] Cianfoni A., Law M., Re T.J., Dubowitz D.J., Rumboldt Z., Imbesi S.G. (2011). Clinical pitfalls related to short and long echo times in cerebral MR spectroscopy. Journal of Neuroradiology.

[48] Schneider JF (2016). MR spectroscopy in children: protocols and pitfalls in non-tumorous brain pathology. Pediatr Radiol..

[49] Reichenbach JR, Essig M, Haacke EM, Lee BC, Przetak C, Kaiser WA (1998). High-resolution venography of the brain using magnetic resonance imaging. MAGMA..

[50] Liu Saifeng, Buch Sagar, Chen Yongsheng, Choi Hyun-Seok, Dai Yongming, Habib Charbel, Hu Jiani, Jung Joon-Yong, Luo Yu, Utriainen David, Wang Meiyun, Wu Dongmei, Xia Shuang, Haacke E. Mark (2016). Susceptibility-weighted imaging: current status and future directions. NMR in Biomedicine.

[51] Soman S, Holdsworth SJ, Barnes PD, Rosenberg J, Andre JB, Bammer R (2013). Improved T2* imaging without increase in scan time: SWI processing of 2D gradient echo. AJNR Am J Neuroradiol..

[52] Bosemani Thangamadhan, Verschuuren Sylvia I., Poretti Andrea, Huisman Thierry A.G.M. (2013). Pitfalls in Susceptibility-Weighted Imaging of the Pediatric Brain. Journal of Neuroimaging.

[53] Zhou J, Payen J-F, Wilson DA, Traystman RJ, van Zijl PCM (2003). Using the amide proton signals of intracellular proteins and peptides to detect pH effects in MRI. Nat Med..

[54] Ward KM, Aletras AH, Balaban RS (2000). A new class of contrast agents for mri based on proton chemical exchange dependent saturation transfer (CEST). J Magn Reson..

[55] Zhou Jinyuan, Heo Hye-Young, Knutsson Linda, van Zijl Peter C.M., Jiang Shanshan (2019). APT-weighted MRI: Techniques, current neuro applications, and challenging issues. Journal of Magnetic Resonance Imaging.

[56] Kamimura K, Nakajo M, Yoneyama T, Takumi K, Kumagae Y, Fukukura Y (2019). Amide proton transfer imaging of tumors: theory, clinical applications, pitfalls, and future directions. Jpn J Radiol..

[57] Thomalla G, Simonsen CZ, Boutitie F, Andersen G, Berthezene Y, Cheng B (2018). MRI-guided thrombolysis for stroke with unknown time of onset. N Engl J Med..

[58] Luo G, Mo D, Tong X, Liebeskind DS, Song L, Ma N (2018). Factors associated with 90-day outcomes of patients with acute posterior circulation stroke treated by mechanical thrombectomy. World Neurosurg..

[59] Bivard A, Krishnamurthy V, Stanwell P, Levi C, Spratt NJ, Davis S (2014). Arterial spin labeling versus bolus-tracking perfusion in hyperacute stroke. Stroke..

[60] Yu S, Ma SJ, Liebeskind DS, Yu D, Li N, Qiao XJ (2018). ASPECTS-based reperfusion status on arterial spin labeling is associated with clinical outcome in acute ischemic stroke patients. J Cereb Blood Flow Metab..

[61] Park M-G, Yoon CH, Baik SK, Park K-P (2015). Susceptibility vessel sign for intra-arterial thrombus in acute posterior cerebral artery infarction. J Stroke Cerebrovasc Dis..

[62] Radbruch A, Mucke J, Schweser F, Deistung A, Ringleb PA, Ziener CH (2013). Comparison of susceptibility weighted imaging and TOF-angiography for the detection of Thrombi in acute stroke. PLoS One..

[63] Dhundass S, Savatovsky J, Duron L et al (2019) Improved detection and characterization of arterial occlusion in acute ischemic stroke using Contrast Enhanced MRA. J Neuroradiol S0150-9861(19)30170–30171. 10.1016/j.neurad.2019.02.011 10.1016/j.neurad.2019.02.011 30853544

[64] Hodel J, Leclerc X, Kalsoum E, Zuber M, Tamazyan R, Benadjaoud MA (2017). Intracranial arteriovenous shunting: detection with arterial spin-labeling and susceptibility-weighted imaging combined. AJNR Am J Neuroradiol..

[65] Jensen-Kondering U, Lindner T, van Osch MJP, Rohr A, Jansen O, Helle M (2015). Superselective pseudo-continuous arterial spin labeling angiography. Eur J Radiol..

[66] Sunwoo L, Sohn C-H, Lee JY, Yi KS, Yun TJ, Choi SH (2015). Evaluation of the degree of arteriovenous shunting in intracranial arteriovenous malformations using pseudo-continuous arterial spin labeling magnetic resonance imaging. Neuroradiology..

[67] Kodera T, Arai Y, Arishima H, Higashino Y, Isozaki M, Tsunetoshi K (2017). Evaluation of obliteration of arteriovenous malformations after stereotactic radiosurgery with arterial spin labeling MR imaging. Br J Neurosurg..

[68] Nabavizadeh SA, Edgar JC, Vossough A (2014). Utility of susceptibility-weighted imaging and arterial spin perfusion imaging in pediatric brain arteriovenous shunting. Neuroradiology..

[69] Iryo Y, Hirai T, Kai Y, Nakamura M, Shigematsu Y, Kitajima M (2014). Intracranial dural arteriovenous fistulas: evaluation with 3-T four-dimensional MR angiography using arterial spin labeling. Radiology..

[70] Cohen Jacob (1960). A Coefficient of Agreement for Nominal Scales. Educational and Psychological Measurement.

[71] Amukotuwa SA, Heit JJ, Marks MP, Fischbein N, Bammer R (2016). Detection of cortical venous drainage and determination of the Borden type of dural arteriovenous fistula by means of 3D pseudocontinuous arterial spin-labeling MRI. AJR Am J Roentgenol..

[72] Edjlali M, Roca P, Rabrait C, Trystram D, Rodriguez-Régent C, Johnson KM (2014). MR selective flow-tracking cartography: a postprocessing procedure applied to four-dimensional flow MR imaging for complete characterization of cranial dural arteriovenous fistulas. Radiology..

[73] Clark Z, Johnson KM, Wu Y, Edjlali M, Mistretta C, Wieben O (2016). Accelerated time-resolved contrast-enhanced magnetic resonance angiography of dural arteriovenous fistulas using highly constrained reconstruction of sparse cerebrovascular data sets. Invest Radiol..

[74] Hiratsuka Y, Miki H, Kiriyama I, Kikuchi K, Takahashi S, Matsubara I (2008). Diagnosis of unruptured intracranial aneurysms: 3T MR angiography versus 64-channel multi-detector row CT angiography. Magn Reson Med Sci..

[75] Hirai T, Korogi Y, Arimura H, Katsuragawa S, Kitajima M, Yamura M (2005). Intracranial aneurysms at MR angiography: effect of computer-aided diagnosis on radiologists’ detection performance. Radiology..

[76] Chng SM, Petersen ET, Zimine I, Sitoh Y-Y, Lim CCT, Golay X (2008). Territorial arterial spin labeling in the assessment of collateral circulation: comparison with digital subtraction angiography. Stroke..

[77] Iryo Y, Hirai T, Nakamura M, Inoue Y, Watanabe M, Ando Y (2015). Collateral circulation via the circle of Willis in patients with carotid artery steno-occlusive disease: evaluation on 3-T 4D MRA using arterial spin labelling. Clin Radiol..

[78] Ito K, Sasaki M, Kobayashi M, Ogasawara K, Nishihara T, Takahashi T (2014). Noninvasive evaluation of collateral blood flow through circle of willis in cervical carotid stenosis using selective magnetic resonance angiography. J Stroke Cerebrovasc Dis..

[79] Uchino H, Ito M, Fujima N, Kazumata K, Yamazaki K, Nakayama N (2015). A novel application of four-dimensional magnetic resonance angiography using an arterial spin labeling technique for noninvasive diagnosis of moyamoya disease. Clin Neurol Neurosurg..

[80] Sugino T, Mikami T, Miyata K, Suzuki K, Houkin K, Mikuni N (2013). Arterial spin-labeling magnetic resonance imaging after revascularization of moyamoya disease. J Stroke Cerebrovasc Dis..

[81] Noguchi T, Kawashima M, Nishihara M, Egashira Y, Azama S, Irie H (2015). Noninvasive method for mapping CVR in moyamoya disease using ASL-MRI. Eur J Radiol..

[82] Lee S, Yun TJ, Yoo R-E, Yoon B-W, Kang KM, Choi SH et al (2018) Monitoring cerebral perfusion changes after revascularization in patients with moyamoya disease by using arterial spin-labeling MR imaging. Radiology. 170509 10.1148/radiol.2018170509 29714677

[83] Salmela MB, Mortazavi S, Jagadeesan BD, Broderick DF, Burns J, Expert Panel on Neurologic Imaging (2017). ACR appropriateness criteria cerebrovascular disease. J Am Coll Radiol..

[84] Ferro JM, Bousser M-G, Canhão P, Coutinho JM, Crassard I, Dentali F (2017). European Stroke Organization guideline for the diagnosis and treatment of cerebral venous thrombosis - endorsed by the European Academy of Neurology. Eur J Neurol..

[85] Niu P-P, Yu Y, Guo Z-N, Jin H, Liu Y, Zhou H-W (2016). Diagnosis of non-acute cerebral venous thrombosis with 3D T1-weighted black blood sequence at 3T. J Neurol Sci..

[86] Renard D, Le Bars E, Arquizan C, Gaillard N, de Champfleur NM, Mourand I (2017). Time-of-flight MR angiography in cerebral venous sinus thrombosis. Acta Neurol Belg.

[87] Ozturk K, Soylu E, Parlak M (2018) Dural venous sinus thrombosis: The combination of noncontrast CT, MRI and PC-MR venography to enhance accuracy. Neuroradiol J. 1971400918781969 10.1177/1971400918781969 PMC6136129 29869561

[88] Tubridy N, Molyneux PD, Moseley IF, Miller DH (1999). The sensitivity of thin-slice fast spin echo, fast FLAIR and gadolinium-enhanced T1-weighted MRI sequences in detecting new lesion activity in multiple sclerosis. J Neurol.

[89] Thompson AJ, Banwell BL, Barkhof F, Carroll WM, Coetzee T, Comi G (2018). Diagnosis of multiple sclerosis: 2017 revisions of the McDonald criteria. Lancet Neurol.

[90] Geraldes R, Ciccarelli O, Barkhof F, De Stefano N, Enzinger C, Filippi M (2018). The current role of MRI in differentiating multiple sclerosis from its imaging mimics. Nat Rev Neurol.

[91] Traboulsee A, Simon JH, Stone L, Fisher E, Jones DE, Malhotra A (2016). Revised recommendations of the consortium of MS centers task force for a standardized mri protocol and clinical guidelines for the diagnosis and follow-up of multiple sclerosis. AJNR Am J Neuroradiol.

[92] Held U, Heigenhauser L, Shang C, Kappos L, Polman C, Sylvia Lawry Centre for MS Research (2005). Predictors of relapse rate in MS clinical trials. Neurology..

[93] Daumer M, Neuhaus A, Morrissey S, Hintzen R, Ebers GC (2009). MRI as an outcome in multiple sclerosis clinical trials. Neurology..

[94] Shinohara RT, Goldsmith J, Mateen FJ, Crainiceanu C, Reich DS (2012). Predicting breakdown of the blood-brain barrier in multiple sclerosis without contrast agents. AJNR Am J Neuroradiol..

[95] Barkhof F, Held U, Simon JH, Daumer M, Fazekas F, Filippi M (2005). Predicting gadolinium enhancement status in MS patients eligible for randomized clinical trials. Neurology..

[96] Gupta A, Al-Dasuqi K, Xia F, Askin G, Zhao Y, Delgado D (2017). The use of noncontrast quantitative MRI to detect gadolinium-enhancing multiple sclerosis brain lesions: a systematic review and meta-analysis. AJNR Am J Neuroradiol..

[97] Michoux N, Guillet A, Rommel D, Mazzamuto G, Sindic C, Duprez T (2015). Texture analysis of T2-weighted MR images to assess acute inflammation in brain MS lesions. PLoS One..

[98] Jurcoane A, Wagner M, Schmidt C, Mayer C, Gracien R-M, Hirschmann M (2013). Within-lesion differences in quantitative MRI parameters predict contrast enhancement in multiple sclerosis. J Magn Reson Imaging..

[99] Popescu V, Agosta F, Hulst HE, Sluimer IC, Knol DL, Sormani MP (2013). Brain atrophy and lesion load predict long term disability in multiple sclerosis. J Neurol Neurosurg Psychiatry..

[100] Brex PA, Ciccarelli O, O’Riordan JI, Sailer M, Thompson AJ, Miller DH (2002). A longitudinal study of abnormalities on MRI and disability from multiple sclerosis. N Engl J Med..

[101] Granberg T, Uppman M, Hashim F, Cananau C, Nordin LE, Shams S (2016). Clinical feasibility of synthetic MRI in multiple sclerosis: a diagnostic and volumetric validation study. AJNR Am J Neuroradiol..

[102] Berry I, Brant-Zawadzki M, Osaki L, Brasch R, Murovic J, Newton TH (1986). Gd-DTPA in clinical MR of the brain: 2. Extraaxial lesions and normal structures. AJR Am J Roentgenol..

[103] Brant-Zawadzki M, Berry I, Osaki L, Brasch R, Murovic J, Norman D (1986). Gd-DTPA in clinical MR of the brain: 1. Intraaxial lesions. American Journal of Roentgenology.

[104] Verburg N, Hoefnagels FWA, Barkhof F, Boellaard R, Goldman S, Guo J (2017). Diagnostic accuracy of neuroimaging to delineate diffuse gliomas within the brain: a meta-analysis. AJNR Am J Neuroradiol..

[105] Boonzaier NR, Larkin TJ, Matys T, van der Hoorn A, Yan J-L, Price SJ (2017). Multiparametric MR imaging of diffusion and perfusion in contrast-enhancing and nonenhancing components in patients with glioblastoma. Radiology..

[106] Goebell E, Paustenbach S, Vaeterlein O, Ding X-Q, Heese O, Fiehler J (2006). Low-grade and anaplastic gliomas: differences in architecture evaluated with diffusion-tensor MR imaging. Radiology..

[107] Lu S, Ahn D, Johnson G, Law M, Zagzag D, Grossman RI (2004). Diffusion-tensor MR imaging of intracranial neoplasia and associated peritumoral edema: introduction of the tumor infiltration index. Radiology..

[108] Fink KR, Fink JR (2013). Imaging of brain metastases. Surg Neurol Int..

[109] Tomura N, Narita K, Takahashi S, Otani T, Sakuma I, Yasuda K (2007). Contrast-enhanced multi-shot echo-planar FLAIR in the depiction of metastatic tumors of the brain: comparison with contrast-enhanced spin-echo T1-weighted imaging. Acta radiol.

[110] Ahn SJ, Chung T-S, Chang J-H, Lee S-K (2014). The added value of double dose gadolinium enhanced 3D T2 fluid-attenuated inversion recovery for evaluating small brain metastases. Yonsei Med J..

[111] Chamberlain M, Junck L, Brandsma D, Soffietti R, Rudà R, Raizer J (2017). Leptomeningeal metastases: a RANO proposal for response criteria. Neuro Oncol.

[112] Rodriguez Gutierrez D, Awwad A, Meijer L, Manita M, Jaspan T, Dineen RA (2014). Metrics and textural features of MRI diffusion to improve classification of pediatric posterior fossa tumors. AJNR Am J Neuroradiol.

[113] Fetit AE, Novak J, Peet AC, Arvanitits TN (2015). Three-dimensional textural features of conventional MRI improve diagnostic classification of childhood brain tumours. NMR Biomed..

[114] Ko CC, Tai MH, Li CF, Chen TY, Chen JH, Shu G (2016). Differentiation between glioblastoma multiforme and primary cerebral lymphoma: additional benefits of quantitative diffusion-weighted MR imaging. PLoS One..

[115] You Sung-Hye, Yun Tae Jin, Choi Hye Jeong, Yoo Roh-Eul, Kang Koung Mi, Choi Seung Hong, Kim Ji-hoon, Sohn Chul-Ho (2018). Differentiation between primary CNS lymphoma and glioblastoma: qualitative and quantitative analysis using arterial spin labeling MR imaging. European Radiology.

[116] Vallée A, Guillevin C, Wager M, Delwail V, Guillevin R, Vallée J-N (2018) Added value of spectroscopy to perfusion MRI in the differential diagnostic performance of common malignant brain tumors. AJNR Am J Neuroradiol. 10.3174/ajnr.A5725 10.3174/ajnr.A5725 PMC7410531 30049719

[117] Sunwoo L, Yun TJ, You S-H, Yoo R-E, Kang KM, Choi SH (2016). Differentiation of glioblastoma from brain metastasis: qualitative and quantitative analysis using arterial spin labeling MR imaging. PLoS One.

[118] Badve C, Yu A, Dastmalchian S, Rogers M, Ma D, Jiang Y (2017). MR fingerprinting of adult brain tumors: initial experience. AJNR Am J Neuroradiol..

[119] Tan Y, Wang X-C, Zhang H, Wang J, Qin J-B, Wu X-F (2015). Differentiation of high-grade-astrocytomas from solitary-brain-metastases: comparing diffusion kurtosis imaging and diffusion tensor imaging. Eur J Radiol.

[120] Neska-Matuszewska M, Bladowska J, Sąsiadek M, Zimny A (2018). Differentiation of glioblastoma multiforme, metastases and primary central nervous system lymphomas using multiparametric perfusion and diffusion MR imaging of a tumor core and a peritumoral zone-Searching for a practical approach. PLoS One..

[121] Sakata A, Fushimi Y, Okada T, Arakawa Y, Kunieda T, Minamiguchi S (2017). Diagnostic performance between contrast enhancement, proton MR spectroscopy, and amide proton transfer imaging in patients with brain tumors. J Magn Reson Imaging..

[122] Usinskiene J, Ulyte A, Bjørnerud A, Venius J, Katsaros VK, Rynkeviciene R (2016). Optimal differentiation of high- and low-grade glioma and metastasis: a meta-analysis of perfusion, diffusion, and spectroscopy metrics. Neuroradiology..

[123] Park JE, Kim HS, Park KJ, Choi CG, Kim SJ (2015). Histogram analysis of amide proton transfer imaging to identify contrast-enhancing low-grade brain tumor that mimics high-grade tumor: increased accuracy of MR perfusion. Radiology..

[124] Falk Delgado A, Nilsson M, van Westen D, Falk Delgado A (2018). Glioma grade discrimination with MR diffusion kurtosis imaging: a meta-analysis of diagnostic accuracy. Radiology..

[125] Hu Y-C, Yan L-F, Wu L, Du P, Chen B-Y, Wang L (2014). Intravoxel incoherent motion diffusion-weighted MR imaging of gliomas: efficacy in preoperative grading. Sci Rep..

[126] Lin NU, Lee EQ, Aoyama H, Barani IJ, Barboriak DP, Baumert BG (2015). Response assessment criteria for brain metastases: proposal from the RANO group. Lancet Oncol..

[127] Park JE, Kim HS, Park KJ, Kim SJ, Kim JH, Smith SA (2016). Pre- and posttreatment glioma: comparison of amide proton transfer imaging with MR spectroscopy for biomarkers of tumor proliferation. Radiology..

[128] Park KJ, Kim HS, Park JE, Shim WH, Kim SJ, Smith SA (2016). Added value of amide proton transfer imaging to conventional and perfusion MR imaging for evaluating the treatment response of newly diagnosed glioblastoma. Eur Radiol..

[129] Wang S, Martinez-Lage M, Sakai Y, Chawla S, Kim SG, Alonso-Basanta M (2016). Differentiating tumor progression from pseudoprogression in patients with glioblastomas using diffusion tensor imaging and dynamic susceptibility contrast MRI. AJNR Am J Neuroradiol..

[130] Fink JR, Carr RB, Matsusue E, Iyer RS, Rockhill JK, Haynor DR (2012). Comparison of 3 Tesla proton MR spectroscopy, MR perfusion and MR diffusion for distinguishing glioma recurrence from posttreatment effects. J Magn Reson Imaging..

[131] Lescher S, Jurcoane A, Veit A, Bähr O, Deichmann R, Hattingen E (2015). Quantitative T1 and T2 mapping in recurrent glioblastomas under bevacizumab: earlier detection of tumor progression compared to conventional MRI. Neuroradiology..

[132] Tiwari P, Prasanna P, Wolansky L, Pinho M, Cohen M, Nayate AP (2016). Computer-extracted texture features to distinguish cerebral radionecrosis from recurrent brain tumors on multiparametric MRI: a feasibility study. AJNR Am J Neuroradiol..

[133] Weybright P, Maly P, Gomez-Hassan D, Blaesing C, Sundgren PC (2004). MR spectroscopy in the evaluation of recurrent contrast-enhancing lesions in the posterior fossa after tumor treatment. Neuroradiology..

[134] Kimura T, Sako K, Tanaka K, Gotoh T, Yoshida H, Aburano T (2004). Evaluation of the response of metastatic brain tumors to stereotactic radiosurgery by proton magnetic resonance spectroscopy, 201TlCl single-photon emission computerized tomography, and gadolinium-enhanced magnetic resonance imaging. J Neurosurg.

[135] Zeng Q-S, Li C-F, Zhang K, Liu H, Kang X-S, Zhen J-H (2007). Multivoxel 3D proton MR spectroscopy in the distinction of recurrent glioma from radiation injury. J Neurooncol..

[136] Schlemmer HP, Bachert P, Herfarth KK, Zuna I, Debus J, van Kaick G (2001). Proton MR spectroscopic evaluation of suspicious brain lesions after stereotactic radiotherapy. AJNR Am J Neuroradiol..

[137] Calmon R, Puget S, Varlet P, Dangouloff-Ros V, Blauwblomme T, Beccaria K (2018). Cerebral blood flow changes after radiation therapy identifies pseudoprogression in diffuse intrinsic pontine gliomas. Neuro Oncol..

[138] Suh Chong Hyun, Kim Ho Sung, Jung Seung Chai, Choi Choong Gon, Kim Sang Joon (2018). Multiparametric MRI as a potential surrogate endpoint for decision-making in early treatment response following concurrent chemoradiotherapy in patients with newly diagnosed glioblastoma: a systematic review and meta-analysis. European Radiology.

[139] Nadal Desbarats L, Herlidou S, de Marco G, Gondry-Jouet C, Le Gars D, Deramond H (2003). Differential MRI diagnosis between brain abscesses and necrotic or cystic brain tumors using the apparent diffusion coefficient and normalized diffusion-weighted images. Magn Reson Imaging..

[140] Fertikh Djamil, Krejza Jaroslaw, Cunqueiro Alain, Danish Shabbar, Alokaili Riyadh, Melhem Elias R. (2007). Discrimination of capsular stage brain abscesses from necrotic or cystic neoplasms using diffusion-weighted magnetic resonance imaging. Journal of Neurosurgery.

[141] Splendiani A, Puglielli E, De Amicis R, Necozione S, Masciocchi C, Gallucci M (2005). Contrast-enhanced FLAIR in the early diagnosis of infectious meningitis. Neuroradiology..

[142] Fukuoka H, Hirai T, Okuda T, Shigematsu Y, Sasao A, Kimura E (2010). Comparison of the added value of contrast-enhanced 3D fluid-attenuated inversion recovery and magnetization-prepared rapid acquisition of gradient echo sequences in relation to conventional postcontrast T1-weighted images for the evaluation of leptomeningeal diseases at 3T. AJNR Am J Neuroradiol..

[143] Hong JT, Son BC, Sung JH, Kim IS, Yang SH, Lee SW (2008). Significance of diffusion-weighted imaging and apparent diffusion coefficient maps for the evaluation of pyogenic ventriculitis. Clin Neurol Neurosurg..

[144] Miki Yukio, Isoda Hiroyoshi, Togashi Kaori (2009). Guideline to use gadolinium-based contrast agents at Kyoto University Hospital. Journal of Magnetic Resonance Imaging.

[145] Schieda Nicola, Blaichman Jason I., Costa Andreu F., Glikstein Rafael, Hurrell Casey, James Matthew, Jabehdar Maralani Pejman, Shabana Wael, Tang An, Tsampalieros Anne, van der Pol Christian B., Hiremath Swapnil (2018). Gadolinium-Based Contrast Agents in Kidney Disease: A Comprehensive Review and Clinical Practice Guideline Issued by the Canadian Association of Radiologists. Canadian Journal of Kidney Health and Disease.

[146] Costa AF, van der Pol CB, Maralani PJ, McInnes MDF, Shewchuk JR, Verma R (2018). Gadolinium deposition in the brain: a systematic review of existing guidelines and policy statement issued by the Canadian Association of Radiologists. Can Assoc Radiol J..

[147] Center for Drug Evaluation, Research Drug safety and availability - FDA drug safety communication: FDA evaluating the risk of brain deposits with repeated use of gadolinium-based contrast agents for magnetic resonance imaging (MRI). Center for Drug Evaluation and Research; Available https://www.fda.gov/Drugs/DrugSafety/ucm455386.htm

[148] EMA (2017) EMA’s final opinion confirms restrictions on use of linear gadolinium agents in body scans. Available via https://www.ema.europa.eu/documents/referral/gadolinium-article-31-referral-emas-final-opinion-confirms-restrictions-use-linear-gadoliniumagents_en.pdf. Accessed on 9 Dec 2018

[149] Hopewell S, Clarke M, Stewart L, Tierney J (2007) Time to publication for results of clinical trials. Cochrane Database Syst Rev 18;(2):MR000011. 10.1002/14651858.MR000011.pub2 PMC7437393 17443632

[150] Ray JG, Vermeulen MJ, Bharatha A, Montanera WJ, Park AL (2016). Association between MRI exposure during pregnancy and fetal and childhood outcomes. JAMA..

